# miR-27b Modulates Insulin Signaling in Hepatocytes by Regulating Insulin Receptor Expression

**DOI:** 10.3390/ijms21228675

**Published:** 2020-11-17

**Authors:** Asier Benito-Vicente, Kepa B. Uribe, Noemi Rotllan, Cristina M. Ramírez, Shifa Jebari-Benslaiman, Leigh Goedeke, Alberto Canfrán-Duque, Unai Galicia-García, Diego Saenz De Urturi, Patricia Aspichueta, Yajaira Suárez, Carlos Fernández-Hernando, Cesar Martín

**Affiliations:** 1 Biofisika Institute (UPV/EHU, CSIC) and Departamento de Bioquímica, Universidad del País Vasco, 48940 Leioa, Spain; asierbenitovicente@gmail.com (A.B.-V.); kbelloso@cicbiomagune.es (K.B.U.); shifajebari@gmail.com (S.J.-B.); u.galiciag@gmail.com (U.G.-G.); 2 Vascular Biology and Therapeutics Program, Integrative Cell Signaling and Neurobiology of Metabolism Program, Department of Comparative Medicine and Department of Pathology, Yale University School of Medicine, New Haven, CT 06520-8066, USA; NRotllanV@santpau.cat (N.R.); cristina.ramirez@imdea.org (C.M.R.); leigh.goedeke@yale.edu (L.G.); alberto.canfran@yale.edu (A.C.-D.); yajaira.suarez@yale.edu (Y.S.); 3 IMDEA Research Institute of Food and Health Sciences, 28049 Madrid, Spain; 4 Fundación Biofisika Bizkaia, 48940 Leioa, Spain; 5 Department of Physiology, Faculty of Medicine and Nursing, University of Basque Country UPV/EHU, 48940 Leioa, Spain; diego.saenzdeurturi@ehu.eus (D.S.D.U.); patricia.aspichueta@ehu.eus (P.A.)

**Keywords:** microRNA, type 2 diabetes mellitus, miR-27b, insulin signaling, INRS

## Abstract

Insulin resistance (IR) is one of the key contributing factors in the development of type 2 diabetes mellitus (T2DM). However, the molecular mechanisms leading to IR are still unclear. The implication of microRNAs (miRNAs) in the pathophysiology of multiple cardiometabolic pathologies, including obesity, atherosclerotic heart failure and IR, has emerged as a major focus of interest in recent years. Indeed, upregulation of several miRNAs has been associated with obesity and IR. Among them, miR-27b is overexpressed in the liver in patients with obesity, but its role in IR has not yet been thoroughly explored. In this study, we investigated the role of miR-27b in regulating insulin signaling in hepatocytes, both in vitro and in vivo. Therefore, assessment of the impact of miR-27b on insulin resistance through the hepatic tissue is of special importance due to the high expression of miR-27b in the liver together with its known role in regulating lipid metabolism. Notably, we found that miR-27b controls post-transcriptional expression of numerous components of the insulin signaling pathway including the insulin receptor (INSR) and insulin receptor substrate 1 (IRS1) in human hepatoma cells. These results were further confirmed in vivo showing that overexpression and inhibition of hepatic miR-27 enhances and suppresses hepatic INSR expression and insulin sensitivity, respectively. This study identified a novel role for miR-27 in regulating insulin signaling, and this finding suggests that elevated miR-27 levels may contribute to early development of hepatic insulin resistance.

## 1. Introduction

With more than 1.9 billion overweight adults and 650 million obese people in 2016, obesity has become a global epidemic that has nearly tripled since 1975 [1]. Obesity is associated with increased risk for cardiovascular disease, type 2 diabetes mellitus (T2DM), hypertension and coronary heart disease. In particular, obesity-related T2DM is expected to double in prevalence to 300 million by 2025 [2]. Although the molecular mechanisms leading to obesity-related T2DM remain unclear, common features in obese individuals with T2DM include elevation of triglycerides (TG), reduction in high-density lipoprotein (HDL)-C, increased concentration of ApoB-100, small dense low-density lipoprotein (LDL) and HDL.

Obesity leads to increased circulating lipids (cholesterol and TG) that may promote excess lipid deposition in the heart, muscle, pancreas or liver [3]. In the liver, the imbalance among lipid synthesis, uptake, secretion and oxidation results in deleterious lipid accumulation, which results in non-alcoholic fatty liver disease (NAFLD) [4]. Hepatocyte lipotoxicity can activate cell death and endoplasmic reticulum stress response pathway, thereby inducing activation of Kupffer cells and recruitment of extra-hepatic monocytes/macrophages, leading to hepatic inflammation. Eventually, liver lipid overload and increased inflammation promote liver insulin resistance (IR) [5,6], characterized by impaired insulin signaling. Under these circumstances, insulin exerts a lower biological effect than the expected [7] and consequently IR constitutes one of the major factors for developing T2DM. Furthermore, IR is commonly associated with NAFLD or cardiovascular disease (CVD) [7,8,9], which in turn have been associated with changes in lipid and lipoprotein metabolism that could promote both pathogenic situations [10].

Under physiological conditions, insulin binds to insulin receptor (INSR), which triggers INSR dimerization and autophosphorylation. This event leads to phosphorylation of INRS substrates (IRS), as well as other proteins that, in turn, activate multiple intracellular signaling intermediates. In particular, INSR/IRS phosphorylation promotes Phosphatidylinositol 3-kinase/Protein kinase B (PI3K/AKT) pathway activation. In fact, AKT phosphorylation is a major factor in insulin signaling as it promotes glucose uptake [11], glycogen synthesis activation [12] and gluconeogenesis inhibition [13,14]. Activation of insulin signaling via IRS/PI3-kinase pathway is especially relevant in liver tissue. Under high glucose concentrations, insulin reduces hepatic glucose de novo synthesis, enhances glycogen synthesis and glucose uptake and promotes lipid synthesis in order to reduce plasma glucose levels. However, in IR animal models and in vitro models [15] insulin target tissues (liver, muscle or adipose tissue) show reduced glucose uptake and glycogen synthesis and enhanced glucose de novo synthesis, thus contributing to aggravation of hyperglycemia [16]. During the initial stages of IR and T2DM, pancreatic β-cells increase insulin production to compensate for high circulating glucose levels [17]. However, as the disease progresses, the cells become unable to respond to high glucose levels and, in turn, reduce insulin secretion. Under these circumstances, the combination of diminished insulin production along with reduced insulin sensitivity leads to hyperglycemia, which is a hallmark of the IR to T2DM transition [17,18].

To date, multiple key processes underlying the molecular mechanism leading to IR, which include polymorphisms in insulin cascade-related genes, have been identified [8,19]. However, post-transcriptional mechanisms and their role in IR pathogenesis remain less well characterized. Recently, a major focus of interest has emerged on the role of microRNAs (miRNAs) as regulators of the pathophysiology of multiple cardiometabolic pathologies, including obesity, IR, atherosclerosis and heart failure [20,21,22,23,24]. There is evidence that the coordinated action of multiple miRNAs regulates multiple pathways that may converge to promote development of IR [25,26,27,28,29], T2DM and CVD [30,31,32]. Therefore, gaining insight into miRNA-mediated IR development may provide enormous potential in the prevention and early diagnosis of disease. Upregulation of several miRNAs has been associated with both obesity and IR, including miR-27b which has been shown to be overexpressed in the liver of obese people [33,34]. miR-27b controls the expression of genes regulating hepatic lipid metabolism including angiopoietin-like 3 (ANGPTL3) and glycerol-3-phosphate acyltransferase 1, mitochondrial (GPAM) [33,34]. While the role of miR-27b in lipid metabolism is well established, its contribution in regulating insulin signaling in hepatocytes is not known. The high expression levels of miR-27b in the liver, its role in regulation of lipid metabolism and its ~3-fold upregulation determined in liver of mice on a high-fat diet [34] highlight the importance of determining the impact of miR-27b on development IR through hepatic tissue. In addition, given that lipid metabolism alteration is closely related to IR, studying the role of miR-27b in hepatocytes is particularly relevant. In this study, we elucidated how miR-27 expression affects insulin signaling in the liver and its contribution to the development of hepatic insulin resistance in high lipid concentration environments. Notably, we found that miR-27b levels in human hepatoma cell, Huh7, influence the expression of numerous components of the insulin signaling pathways including the INSR and insulin receptor substrate 1 (IRS1). These results were further confirmed in vivo showing that overexpression and inhibition of hepatic miR-27 enhances and suppresses INSR expression in the liver, respectively. Together, this study identified a novel role for miR-27 in regulating insulin signaling and these findings suggest that targeting of miR-27 may be a potential approach to increase insulin sensitivity in obese people in prediabetes stage.

## 2. Results

### 2.1. Identification of miR-27b as a Potential Regulator of the Insulin Signaling Pathway

**Figure 1:**
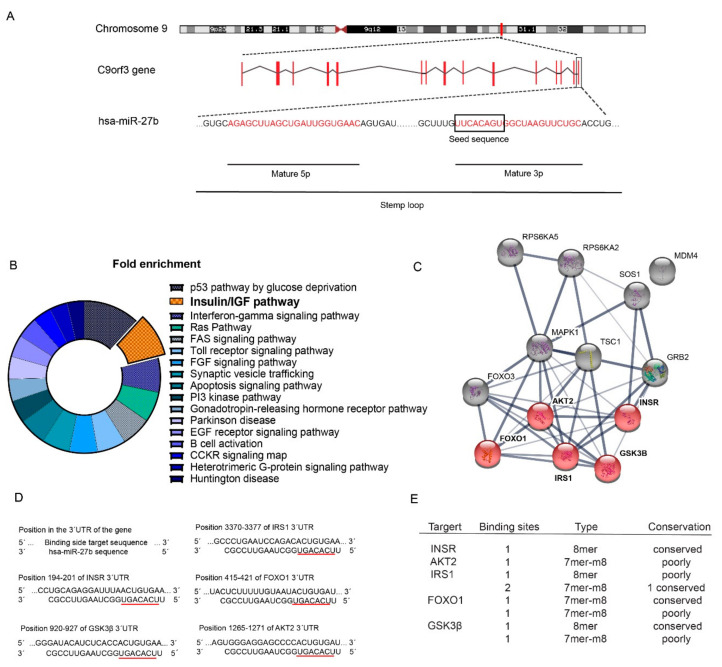
miR-27b is predicted to target many genes involved in insulin signaling pathway. (**A**) schematic representation of miR-27b genomic location; (**B**) pathway enrichment of validated targets of miR-27b; (**C**) interaction gene sequences of the five genes selected in this study with miR-27b; (**D**) genetic representation of miR-27b interactions with the 3′UTR of the selected genes; and (**E**) number, type and conservation of predicted sites of the selected genes.

Previous studies have shown that miR-27b expression is elevated both in diet-induced obese mice and in obese people. Notably, miR-27b is a conserved miR encoded within the 14th intron of *C9orf3* gene (Figure 1A), which is known for regulating lipid-related processes [34] and is involved in cancer regulation [35]. To gain insight into the function of miR-27b, we performed a bioinformatic analysis to identify potential miR-27 target genes using the miRWalk database. These genes were classified according to the pathways in which they are involved (Figure 1B). Interestingly, insulin pathway was one of the pathways overrepresented according to previous studies showing miR-27b overexpression in high fat diet feeding [16,34]. We next performed protein–protein interaction analysis that showed that miR-27b might play an important role in controlling insulin signaling. The genes in the pathway with the highest miR-27 score participating in insulin pathway (*INSR*, *AKT2,* forkhead box protein O1 (*FOXO1*), *IRS1* and glycogen synthase kinase 3 beta (*GSK3B*)) are highlighted in red (Figure 1C). The predicted miR-27b binding sites for these genes are shown in Figure 1D and the number, type and conservation of predicted sites of the selected genes are shown in Figure 1E.

### 2.2. miR-27b Controls the Expression INSR and IRS1 Expression

**Figure 2:**
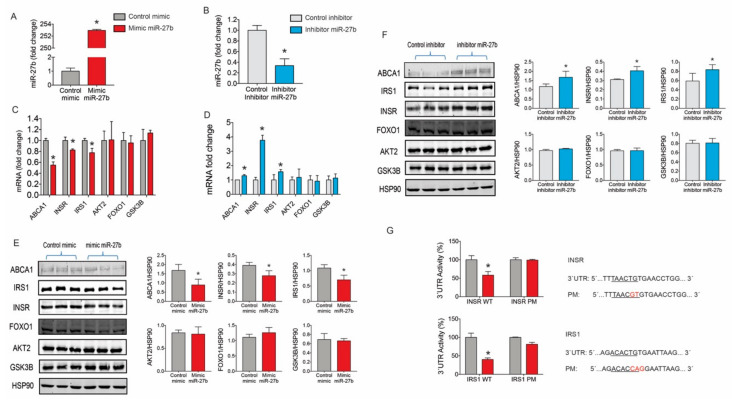
miR-27b modulation affects insulin signaling in Huh7 cell line: (**A**,**B**) miR-27b levels in Huh7 cells transfected with (**A**) 40 nM control mimic and mimic miR-27b or (**B**) 40 nM control inhibitor and inhibitor miR-27b; (**C**,**D**) mRNA expression of *ABCA1*, *INSR*, *IRS1*, *AKT2*, *FOXO1* and *GSK3β* in Huh7 cells transfected with (**C**) 40 nM control mimic and mimic miR-27b or (**D**) 40 nM control inhibitor and inhibitor miR-27b; (**E**) Western blot analysis of ABCA1, INSR, IRS1, AKT2, FOXO1 and GSK3β expression in Huh7 cell transfected with miR-27b mimic or miR-27b inhibitor and their respective controls; (**F**) Western blot analysis of ABCA1, INSR, IRS1, AKT2, FOXO1 and GSK3β expression in Huh7 cell transfected with miR-27b mimic or miR-27b inhibitor and their respective controls; and (**G**) analysis of miR-27b targeting on the 3′UTR of human *INSR* and *IRS1* by luciferase reporter assay. * p <  0.05 (significantly different from cells transfected with the CM or CI). Data are presented as the mean ± SEM. *n* = 3 independent experiments ((A–D,G and right-handed panels of (E,F)). Data correspond to results from a representative experiment among three that gave similar results in left-handed panels of (E,F).

To assess the role of miR-27b in regulating *INSR*, *AKT2, FOXO1 IRS1* and *GSK3B* expression in hepatocytes, we overexpressed or inhibited miR-27 levels in Huh7 cells using miRNA mimics and inhibitors, respectively. Transfection of Huh7 cells with miR-27b mimic and inhibitor markedly increased and decreased miR-27b levels, respectively (Figure 2A,B). Overexpression of miR-27b reduced INSR and IRS1 mRNA and protein levels (Figure 2C,E). Conversely, miR-27b inhibition enhanced the expression of INRS and IRS (Figure 2D,F), indicating a physiological role of miR-27 expression levels in regulating components of insulin signaling pathway. As expected by our previous results, ATP-binding cassette transporter ABCA1 (ABCA1) expression was suppressed and upregulated in Huh7 cells transfected with miR-27b mimics and inhibitors, respectively (Figure 2C–F). Other predicted target genes associated to insulin signaling such as AKT2, FOXO1 or GSK3β were not affected by miR-27b mimic or inhibitor transfection, suggesting that, in these conditions, they are not regulated by miR-27b in Huh7 cells (Figure 2C–F).

**Figure A1:**
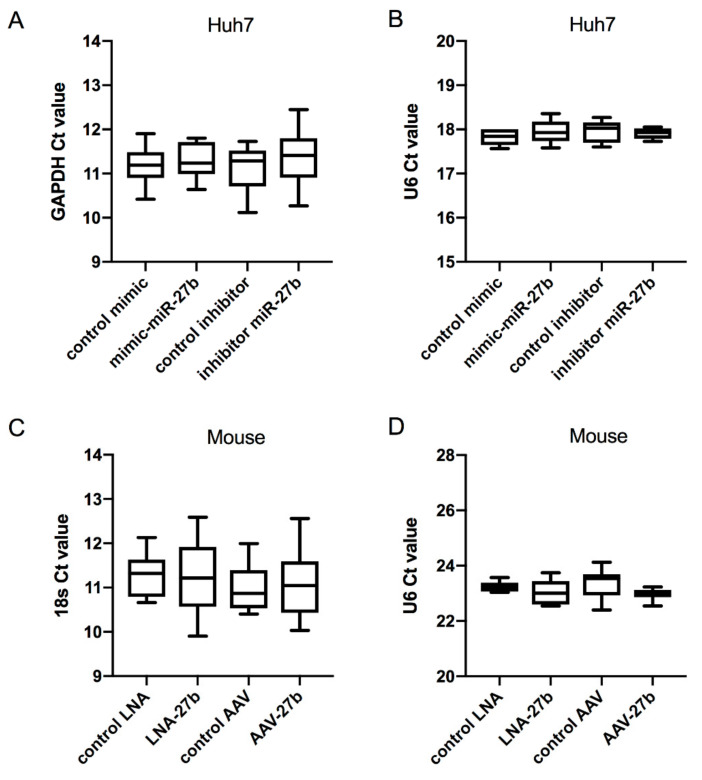
Comparison expression levels of the selected housekeeping RNA molecules: (**A**) GAPDH in Huh7 cells, *p* = 0.204, ANOVA; (**B**) U6 in Huh7 cells, *p* = 0.661; (**C**) 18s in mouse, 18S *p* = 0.464; and (**D**) U6 in mouse, *p* = 0.139. One-way analysis of variance (ANOVA) was used to determine whether a significant difference in GAPDH, 18s and U6 values existed between multiple groups.

There were no significant differences in the expression levels of the selected housekeeping RNA molecules (U6 non-coding small nuclear RNA and glyceraldehyde-3-phosphate dehydrogenase (U6 and GAPDH) for miR and mRNA, respectively; U6 *p* = 0.661 and GAPDH *p* = 0.204; ANOVA) (Figure A1A,B).

To further confirm the specificity of miR-27b targeting on the 3′UTR of human *INSR* and *IRS1*, we used luciferase reporter constructs. miR-27b markedly repressed both *INSR* and *IRS1* 3′UTR activity (Figure 2G). Mutation of the miR-27b target sites relieved miR-27b repression of *INSR* and *IRS1* 3′UTR activity, consistent with a direct interaction of miR-27b with these sites (Figure 2G).

### 2.3. miR-27b Levels Regulate Insulin Signaling in Huh7 Cells

**Figure 3:**
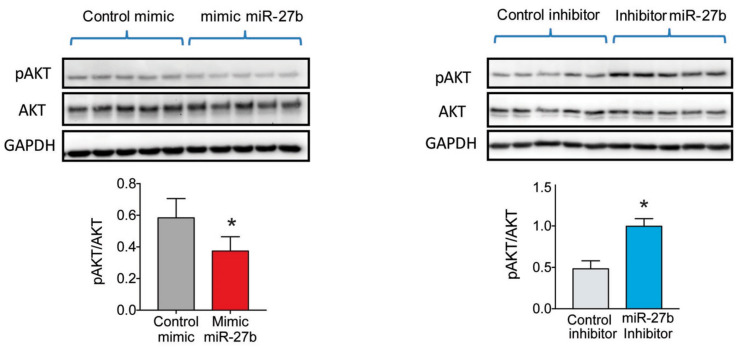
miR-27b levels regulate insulin signaling and attenuates AKT2 phosphorylation in Huh7 cells. Western blot analysis of AKT2 phosphorylation in Huh7 cells treated with miR-27b mimic or miR-27b inhibitor and their respective controls. * p <  0.05 (significantly different from cells transfected with the CM or CI). Data are presented as the mean ± SEM. *n* = 3 independent experiments. Western blot images correspond to results from a representative experiment among three that gave similar results.

We next determined the role of miR-27b expression in regulating insulin signaling in Huh7 cells. To this end, we overexpressed or silenced miR-27b in Huh7 cells and stimulated with insulin. The results show that transfection with miR-27b mimic significantly attenuated AKT2 phosphorylation compared to non-targeted control miRNA (CM) transfected cells. Conversely, inhibition of the endogenous miR-27b increased AKT2 phosphorylation (Figure 3). These results clearly indicate that miR-27b negatively affects insulin signaling when miR-27b is overexpressed and positively affects insulin signaling when miR-27b is inhibited.

### 2.4. miR-27b Regulates Hepatic INSR Expression In Vivo

**Figure 4:**
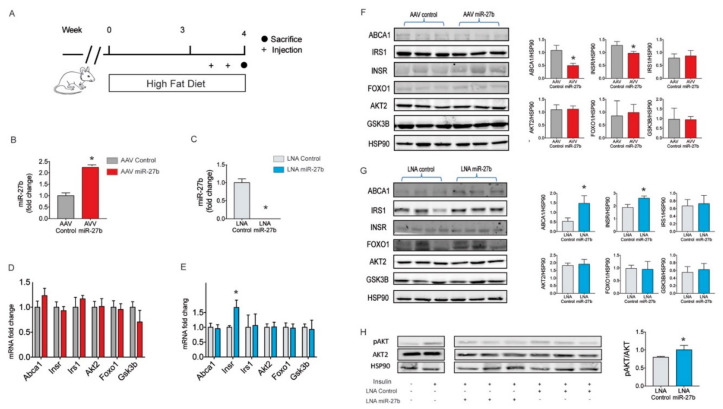
miR-27b modulation affects insulin signaling in wild type mice: (**A**) schematic representation of mice treatment; (**B**,**C**) miR-27b levels in wild type mice treated with (**B**) null AAV or AAV miR-27b or (**C**) LNA control or LNA miR-27b inhibitor; (**D**,**E**) mRNA expression of *Abca1, Insr, Irs1, Akt2, Foxo1* and *Gsk3β* in wild type mice treated with (**D**) null AAV or AAV miR-27b or (**E**) LNA control or LNA miR-27b inhibitor; (**F**) Western blot analysis of ABCA1, INSR, IRS1, AKT2, FOXO1 and GSK3β expression in wild-type mice treated either with AAV-miR-27b or AAV control; (**G**) Western blot analysis of ABCA1, INSR, IRS1, AKT2, FOXO1 and GSK3β expression in wild-type mice treated either with LNA-miR-27b inhibitor or LNA control; and (**H**) Western blot analysis of AKT2 phosphorylation in wild type mice treated with LNA miR-27b inhibitor and its respective control. * p <  0.05 (significantly different from cells transfected with the CM or CI). Data are presented as the mean ± SEM. *n* = 3 independent experiments ((B–E) and right-handed panels of (F–H)). Western blot images correspond to results from a representative experiment among three that gave similar results in (D,G).

Insulin signaling deregulation together with miR-27b upregulation, among others, has been described in mice fed with a HFD [34]. Our previous results showing INSR targeting by miR-27b and the subsequent inhibition of insulin signaling in Huh7 cells suggest that miR-27b might be involved in IR in mice fed a HFD. To test this hypothesis, we overexpressed and silenced miR-27b using an adeno-associated vector (AAV) and LNA, respectively, in mice fed a HFD (to increase the hepatic expression of miR-27b) (Figure 4A). Treatment with AAV-miR-27b resulted in two-fold mature miR-27b enrichment in the liver compared with AAV-control-miR, whereas transfection with LNA anti-miR-27b completely abolished hepatic miR-27b levels (Figure 4B,C). Notably, we found that hepatic INSR expression was markedly downregulated in AAV miR-27b treated mice compared to AAV-Control-miR infected mice through translational repression of *Insr* mRNA instead of mRNA degradation while miR-27b suppression resulted in a significant increase in hepatic INSR expression (Figure 4D–G). As a result, LNA anti-miR-27b treatment enhanced AKT2 phosphorylation upon insulin stimulation (Figure 4H).

There were no significant differences in the expression levels of the selected housekeeping RNA molecules (*U6* and *18s* (18s ribosomal RNA) for miR and mRNA, respectively; *U6 p* = 0.139 and *18s p* = 0.464; ANOVA) (Figure A1C,D).

Taken together, our results indicate that the post-transcriptional regulation mediated by miR-27b both in vitro and in vivo, is able to negatively influence insulin signaling in the liver that may contribute to hepatic IR during the progression to T2DM.

## 3. Discussion

Overweight and obesity are the primary driving forces in T2DM progression [36], which has been continuously rising in developed countries and is therefore predicted to reach epidemic dimensions in the next decades [37]. Obesity is linked to the development of T2DM and cardiovascular diseases possibly through detrimental effects on insulin and glucose metabolism [38]. Indeed, sustained high free fatty acid (FA) plasma concentration, arising from excessive accumulation in adipose tissue in obese people [39], leads to lipid accumulation within hepatocytes. This event is causally related to deregulation of insulin sensitivity in the liver [40].

One of the most relevant pathological characteristics of T2DM is an impaired response to insulin due to disruption of insulin signaling pathway. This disrupted insulin response is especially harmful if developed in certain specific extra pancreatic tissues such as muscle, liver or adipose tissue as they are essential for the maintenance of optimal glycemic homeostasis. As mentioned above, several mechanisms have been described as key modulators of insulin signaling, and, recently, the involvement of specific miRNAs has been described [26,41,42]. Epigenetic studies have emerged as promising candidates to understand the link between obesity and T2DM. Multiple miRNAs acting in concert have been associated with IR in skeletal muscle [43]; however, the role of miRNAs regulating liver insulin signaling is less known. It has been described that, in liver, high plasma lipid levels or HFD induce a three-fold upregulation of miR-27b [34], a well-known regulator of lipid energy homeostasis [44,45,46]. However, there is a gap of information regarding miR-27b implication in liver insulin signaling, even though it has been demonstrated to be upregulated in the liver of obese people [47].

The main focus of interest of the present study has been to elucidate the implication of miR-27b in insulin-dependent liver signaling. miR-27b is encoded in human chromosome 19, specifically in the intron 14 of the *C9orf3* gene as part of the miR-23b-27b-24-1 cluster [34]. The bioinformatic analysis performed in this work highlights the insulin pathway as an enriched target for miR-27b with many proteins potentially regulated by this miRNA. The high interaction score of miR-27b for INSR, IRS1, AKT2, FOXO1 and GSK3β led us next to deeply assess the interaction between miR-27b and its in silico predicted targets both in vitro and in vivo. Accordingly, the data presented here show an inverse correlation between miR-27b expression and both INSR mRNA and protein levels, thus indicating that miR-27b directly regulates INSR in Huh7 cells. Similar to the findings presented here in Huh7 cells, direct downregulation of INSR by miR-27b has also been previously shown in chronic hyperinsulinemia-induced IR adipocytes [48]. Therefore, the results presented here are fully consistent and complement the data reported by Srivastava et al. in adipocytes, which showed miR-27b targeting to the 3′UTR of *INSR*, miR-27b upregulation in insulin-resistant 3T3-L1 adipocytes, direct modulation of insulin signaling by targeting INSR expression and decreased phosphorylation of Akt and AS160 and other parameters of IR [48]. In addition to the miR-27b INSR targeting confirmed in this work, we demonstrate that miR-27b also interferes with insulin signaling by targeting the 3′UTR of *INS1*. Therefore, overexpression of miR-27b negatively regulates IRS1 in Huh7 cells. IRS1 downregulation is normally associated with mild IR due to independent IRS2 compensatory signaling [49]. However, we found that the combination of IRS1 and INSR inhibition generates a strong insulin signaling impairment in Huh7 cells. Together, our data demonstrate the relevance and implications of studying miR-27 expression in a tissue where the abundance of the microRNA is very high. In addition, we show here that in the liver miR-27b not only targets INSR but also INS1, which highlights that hepatic insulin signaling is compromised to a greater extent than in adipocytes. Finally, we identified additional targets of miR-27b related to insulin signaling indicating that insulin signaling is also affected downstream receptor.

Here, we confirm the direct regulation of INSR in the liver in vivo in mice fed a chow diet treated with AAV-27b and mice fed a HFD treated with anti-miR-27b. In agreement with the results obtained with Huh7 cells, miR-27b overexpression diminished INSR protein levels in mice liver, while downregulation of miR-27b caused an increased in INSR expression. In contrast, expression of IRS1 was not modified in mice, neither by miR-27b overexpression nor by its downregulation. The differences observed between the in vitro and in vivo models can be explained by sequence conservation among *IRS1* 3′UTR. While in mice there is only one predicted miR-27b binding site, human *IRS1* 3′UTR has two additional predicted miRNAs binding sites that could be responsible of a more efficient interaction with miR-27b. In fact, the significant lower interaction of miR-27b with mice *IRS1 3′UTR* compared to that determined in humans explains the lack of the effect of miR-27b on IRS1 expression. Nonetheless, we show that INSR downregulation is enough to reduce insulin signaling in vivo.

Hepatic IR is a common feature in obese patients, in which lipid accumulation and chronic inflammation favors its development [5,40]. It is also well established that miRNAs can modulate insulin signaling in the body, although the interplay of different miRNAs leading to IR remains to be elucidated. In vitro and in vivo model systems are the basis for the understanding and control of off-target effects of miRNAs. Modulating the miRNA activities may provide exciting opportunities for obesity and T2DM therapy. To date, several miRNAs are known to be involved in the development of IR through interfering with insulin signaling pathway. For instance, obesity-induced miR-15b and miR-195 overexpression is linked to the pathogenesis of IR in liver by INSR targeting [41,42]. Similar to the miR-27b effects described in this work, miR-15b and miR-195 target *INSR* 3′UTR directly and downregulate INSR expression at a translational level. This direct targeting of *INSR* 3′UTR by different miRNAs that are upregulated in mice fed a HFD suggests that a synergistic effect may potentiate the development of hepatic IR, which in turn may lead to T2DM. As seen in our mice model in which miR-27b does not target IRS1, saturated FA diet-induced overexpression of miR-15b does not suppress IRS1 [41]. In the case of miR-15b, its targeting to INSR results in a drastic repression of protein expression and eventually causes impaired insulin signaling and insulin-stimulated synthesis of glycogen in hepatocytes [41].

There is evidence that certain diabetes-related miRNAs may also play a role in the development of cardiovascular disease and other diabetic complications [50,51]. This suggests that miRNAs may be involved in the interaction between IR and development of additional characteristic cardiometabolic disease traits [52]. miR-128-1, another miRNA affecting the insulin pathway, also directly targets the 3′UTR of the *LDLR* and *ABCA1*, and its inhibition in mice causes a significant drop in circulating cholesterol and TG levels, improved homeostasis and insulin sensitivity by enhancing the expression of INSR and IRS [53]. In other tissues, additional miRNAs are involved in IR development; for example, miR-375 regulates β-cell function and pancreatic insulin secretion [54], miR-135 promotes IR in muscle cells [26] and miR-146a, miR-33 miR-33* and miR-7 impairs insulin signaling downregulating IRS2 in vitro [25,55,56,57,58,59].

The complexity of the miRNAs network that links obesity with T2DM progression led us to focus this study on the effect caused by miR-27b modulation on hepatic insulin signaling. Therefore, INSR was confirmed as metabolic target of miR-27b in both human hepatocarcinoma cell line and mice, thus showing its capacity to initiate IR development at an early stage by modulating INSR expression. Moreover, we found that IRS1 is targeted by miR-27b in human cell line but not in mice due to a lack of conservation in several of the predicted miR-27b binding sites in mice. This effect indicates that, in humans, miR-27b also affects modulation of IR downstream insulin signaling.

This work emphasizes the importance of miRNA modulation studies to determine their functional effects. Specifically, our study demonstrates the direct effect of miR-27b on INSR and IRS1 expression and its potential role as insulin signaling regulator.

## 4. Materials and Methods

### 4.1. Bioinformatics

hsa-miR-27b predicted target genes were identified using the miRWalk 2.0 database (http://www.umm.uni-heidelberg.de/apps/zmf/mirwalk/) which summarizes target interactions from 12 prediction algorithms. For this work, miRWalk, miRanda, RNA22 and TargetScan were specifically used. Only genes matching for the 4 algorithms were introduced in the next step. PANTHER database V.14.0 (http://www.pantherdb.org) was used to classify those genes into different gene/protein families. The 5 genes with the best score among the insulin pathway genes were introduced in STRING 11.0 (http://string-db.org) to visualize functional interactions.

### 4.2. Cell Culture

Huh7 hepatocarcinoma cell line was obtained from American Type Tissue Collection (ATCC, Manassas, VA, USA). Cells were maintained in Dulbecco’s Modified Eagle Medium and 100 U/mL penicillin, 100 μg/mL streptomycin and 4 mM glutamine (DMEM and Pen Strep Glutamine, Merck Life Science, Madrid, Spain) containing 10% (*v*/*v*) fetal bovine serum (FBS) (Lonza, Bornem, Belgium) at 37 °C and 5% CO_2_. For RNA and protein analysis, cells were treated as described below.

### 4.3. In Vitro miRNAs Transfections

Transfection of miR-27b mimic and miR-27b inhibitor was performed in Huh7 cells growing in 6-well culture plates (1 × 10^6^ cells/well) using Lipofectamine™ RNAiMAX Transfection Reagent following manufacturer instructions. Briefly, 10 min before transfection, Huh7 cells were washed with PBS and culture medium was replaced with 500 µL Opti-MEM (Gibco, Madrid, Spain) culture medium without serum and antibiotics. Then, 300 µL transfection mixture was added and plates were incubated for 8 h at 37 °C and 5% CO_2_. Eight hours post transfection, 800 µL of Opti-MEM containing 10% FBS was added to the culture plates and cells were maintained in transfection medium for 48 h to achieve maximum effect. No toxicity was detected 48 h post transfection. Both miR-27b mimic (Dharmacon, Lafayette, CO USA) and miR-27b inhibitor (Dharmacon) were transfected to achieve 40 nM final concentration. All experimental control samples were transfected either with non-targeting control mimic (CM) (Dharmacon) or non-targeting control inhibitor (CI) (Dharmacon).

### 4.4. RNA Isolation and Quantitative PCR Analysis

For mRNA analysis, total RNA was extracted from Huh7 cells transfected with miR-27b mimic, miR-27b inhibitor, CM or CI using TRIzol reagent (Invitrogen, Waltham, MA, USA) according to manufacturer instructions. Extracted RNA (1 µg) was reverse-transcribed using iScript^TM^ Reverse Transcription Supermix (BioRad, Hercules, CA, USA). Quantitative real-time PCR (qRT-PCR) was performed in triplicate using iQ SYBR green Supermix (Bio-Rad) on a Real-Time Detection System (Bio-Rad). The mRNA levels were normalized to ribosomal RNA 18S as a housekeeping gene. Mature miR-27b expression was quantified using TaqMan miRNA Assay kit (Life Technologies, Carlsbad, CA, USA) according to the manufacturer’s instructions. qRT-PCR was performed using TaqMan Universal Master Mix (Life Technologies); U6 RNA was used for normalization. Primer information is detailed in Table A1.

### 4.5. Western Blot Analysis

For Western blot analysis, Huh7 cells transfected with miR-27b mimic, miR-27b inhibitor, CM or CI were lysed using ice-cold buffer containing 50 mM Tris–HCl, 125 mM NaCl, 1% Nonidet P-40, 5.3 mM NaF, 1.5 mM NaP, 1 mM orthovanadate, 1 mg/mL protease inhibitor cocktail (Roche, Basilea, Switzerland) and 0.25 mg/mL Pefabloc, 4-(2-aminoethyl)-benzenesulfonyl fluoride hydrochloride (AEBSF; Roche), pH 7.5. Cells were rotated for 1 h and centrifuged at 12,000× *g* for 10 min. Proteins from the supernatants were resolved by 8.5% Tris-Glycine SDS-PAGE. Gels were then blotted onto nitrocellulose membranes (Protran BA 83, Whatman™; GE Healthcare, Munich, Germany), blocked for 1 h in TBS-T (50 mM Tris-HCl, 150 mM NaCl, pH 7.5, 0.1% Tween 20) containing 5% bovine serum albumin and immunoblotted for 16 h at 4 °C with the following antibodies for 16 h at 4 °C: ABCA1 (1:1000), INSR (1:1000), IRS1 (1:500), AKT2 (1:1000), GSK3β (1:1000), FOXO1 (1:1000) and HSP90 (1:1000) (Table A2). Protein bands were visualized using the Odyssey Infrared Imaging System (LI-COR Biotechnology). Densitometric analysis of the gels was carried out using ImageJ software from the NIH (http://rsbweb.nih.gov/ij/).

### 4.6. 3’UTR Luciferase Reporter Assays

psiCHECK2^TM^ reporter plasmid containing wild type 3′UTR of *IRS1*, *INSR* and their point mutated variants were purchased from GenScript. To carry out the luciferase reporter assay, HEK293 cells were plated into 24-well culture plates and co-transfected with 2 µg of the corresponding 3′UTR luciferase reporter vectors and 40 nM of miR-27b mimic or CM using Lipofectamine 2000 (Invitrogen). Dual-Glo Luciferase Assay system (Promega, Madison, WI, USA) was used to determine luciferase activity. *Renilla* luciferase activity was normalized with that of Firefly luciferase and the results were reported as a percentage of the firefly luciferase activity determined for CM. All the experiments were performed in triplicate in 12-well culture plates and repeated at least three times.

### 4.7. Mice Studies

Eight-week-old male C57BL6 mice (Jackson Laboratories, Bar Harbor, ME, USA) were kept under constant temperature and humidity in a 12 h controlled dark/light cycle [34]. After corresponding treatments described above, liver, adipose and muscle tissue samples were collected as previously described [60] and stored at −80 °C. All mouse experiments were approved by the Institutional Animal Care Use Committee (#2019-11577) of Yale University School of Medicine. 

### 4.8. miR-27b Overexpression Studies in Mice

mmu-pre-miR-27b (accession MI000142) was subcloned into an AAV8 vector and its expression was regulated under the control of a liver-specific thyroxine-binding globulin (TBG) promoter. After 3 weeks in chow diet, mice were divided into 2 groups: non-targeting AAV8 (AAV-Control, *n* = 5) and pre-miR-27b AAV8 (AAV-miR-27b, *n* = 5). Each group was then treated with 5 × 10^12^ GC (genome copies) per kg AAV Null or 5 × 10^12^ GC/kg pre-miR-27b AAV in PBS by intra-peritoneal injection. Two weeks after treatment, mice were sacrificed and hepatic gene expression was analyzed by Western blot and qRT-PCR using the antibodies and primers described in Table A3.

### 4.9. miR-27b Inhibition Studies

Eight-week-old male C57BL6 mice were fed a Western diet ((WD) 9.5% casein, 0.3% DL-methionine, 15% cornstarch, 40% sucrose, 5% cellulose, 21% anhydrous milk fat, 3.5% mineral mix, 1% vitamin mix, 0.4% calcium carbonate and 0.3% cholesterol) for 3 weeks and divided into 2 groups: locked nucleic acid (LNA) control and LNA anti-miR-27b. During the fourth week, both groups were injected twice with 2.5 mg/kg of either LNA control (5′-ACGTGCTATACGCCCA-3′) (*n* = 8) or LNA anti-miR-27b (5′-AACTTAGCCACTGTGA-3′) (*n* = 8) by intra-peritoneal injection. One week after treatment, mice were sacrificed and hepatic gene expression was analyzed by Western blot and qRT-PCR using the antibodies and primers described in Table A4.

### 4.10. Statistical Analyses

Statistical analysis was performed with the SPSS 24.0 and GraphPad Prism 5.01 (GraphPad Software Inc., San Diego, CA, USA). All data are expressed as the mean ± standard deviation (SD). Two-tail Student’s *t*-test was used to compare miR-27b levels, mRNA and protein expression levels and AKT2 phosphorylation in Huh7 cells and in mice. One-way analysis of variance (ANOVA) was used to determine whether a significant difference in GAPDH, 18S and U6 values existed between multiple groups. *p* < 0.05 was considered statistically significant. All probabilities were two-tailed.

## Appendix A

**Table A1:** List of primers used for qRT-PCT analysis in Huh7 cells.

Gene	Forward Primer Sequence (5′ -> 3′)	Reverse Primer Sequence (5′ -> 3′)
*INSR*	AGTTTGAGGACATGGAGAATGTG	ATAGGAACGATCTCTGAACTCCAC
*IRS1*	ACAAACGCTTCTTCGTACTGC	AGTCAGCCCGCTTGTTGATG
*GSK3B*	GGCAGCATGAAAGTTAGCAGA	GGCGACCAGTTCTCCTGAATC
*AKT2*	ACCACAGTCATCGAGAGGACC	GGAGCCACACTTGTAGTCCA
*ABCA1*	ACCCACCCTATGAACAACATGA	GAGTCGGGTAACGGAAACAGG
*GAPDH*	GGAGCGAGATCCCTCCAAAAT	GGCTGTTGTCATACTTCTCATGG

**Table A2:** List of antibodies used for Western blot analysis.

Gene	Antibody	Product Details		Dilution	Host
*INSR*	INSRβ (C18C4)	Novus	NBP1-19192	1/1000	Mouse
*IRS1*	IRS1	Novus	NB100-58837	1/1000	Rabbit
*GSK3B*	GSK-3-β (27C10)	Cell Signaling	9454S	1/1000	Rabbit
*AKT2*	AKT2 (5B5)	Cell Signaling	2965S	1/1000	Rabbit
*ABCA1*	ABCA1	Abcam	ab18180	1/1000	Mouse
*HSP90*	HSP90	Bd Pharmigen	610419	1/1000	Mouse

**Table A3:** List of primers used for QRT-PCT analysis in mice tissue.

Gene	Forward Primer Sequence (5′ -> 3′)	Reverse Primer Sequence (5′ -> 3′)
*Insr*	ATGGGCTTCGGGAGAGGAT	CTTCGGGTCTGGTCTTGAACA
*Irs1*	CGATGGCTTCTCAGACGTG	CAGCCCGCTTGTTGATGTTG
*Gsk3b*	ATGGCAGCAAGGTAACCACAG	TCTCGGTTCTTAAATCGCTTGTC
*Akt2*	ACGTGGTGAATACATCAAGACC	GGGCCTCTCCTTATACCCAAT
*Abca1*	GGTTTGGAGATGGTTATACAATAGTTGT	CCCGGAAACGCAAGTCC
*18s*	TTCCGATAACGAACGAGACTCT	TGGCTGAACGCCACTTGTC

**Table A4:** List of antibodies used for AKT phosphorylation studies.

Gene	Antibody	Product Details		Dilution	Host
*AKT*	AKT (5B5)	Cell Signaling	2965S	1/1000	Rabbit
*pAKT*	pAKT Ser473	Cell Signaling	9271S	1/1000	Rabbit
*HSP90*	HSP90	Bd Pharmigen	610419	1/1000	Mouse
